# The Effect of Periodic Duty Cyclings in Metal-Modulated Epitaxy on GaN:Mg Film

**DOI:** 10.3390/ma16041730

**Published:** 2023-02-20

**Authors:** Jun Fang, Wenxian Yang, Xue Zhang, Aiqin Tian, Shulong Lu, Jianping Liu, Hui Yang

**Affiliations:** 1 School of Nano-Tech and Nano-Bionics, University of Science and Technology of China, Hefei 230026, China; 2 Key Lab of Nanodevices and Applications, Suzhou Institute of Nano-Tech and Nano-Bionics, Chinese Academy of Sciences, Suzhou 215123, China

**Keywords:** metal modulation epitaxy, hole concentrations, GaN:Mg

## Abstract

Metal modulation epitaxy (MME) is a technique in which metal beams (Al, Ga, In, and Mg) are switched on and off in short periods in an RF MBE system while a continuous nitrogen plasma beam is kept on. We systematically studied the effect of periodic duty cycling on the morphology, crystalline quality, Mg doping concentration, and electrical properties of GaN:Mg films grown by MME. When the metal shutter duty cycling is 20 s open/10 s close, the sample has smooth surface with clear steps even with Mg doping concentration higher than 1 × 10^20^ cm^−3^. The RMS roughness is about 0.5 nm. The FWHM of (002) XRD rocking curve is 230 arcsec and the FWHM of (102) XRD rocking curve is 260 arcsec. As result, a hole concentration of 5 × 10^18^ cm^−3^ and a resistivity of 1.5 Ω·cm have been obtained. The hole concentration increases due to the incorporation of surface accumulated Mg dopants into suitable Ga substitutional sites with minimal formation of compensatory defects.

## 1. Introduction

GaN-based materials are widely used in optoelectronics and power electronics devices [1,2,3], but obtaining p-GaN film with high concentration of holes is still an obstacle at present. The main challenges related to p-type doping GaN include: (1) deep acceptor activation energy, as large as 160 meV, leading to only about 1% activation rate of Mg acceptor at 300 K; (2) low solubility, in the range of 1 × 10^20^ cm^−3^ [4]; (3) intrinsic defect compensation, such as the formation energy of nitrogen vacancies in p-type GaN [5] is low; (4) pyramidal extended defects [6], which also lead to compensation in p-type material. Except for the above challenges, it would cause other problems when doping with high magnesium concentrations. In traditional growth techniques such as metal organic chemical vapor deposition (MOCVD) and molecular beam epitaxy (MBE), when high-concentration Mg is doped in MOCVD, Mg precipitation is likely to occur, resulting in inversion domains and extended defects [7,8]. In films grown by MBE, when excess Mg is used, the films transform from Ga polarity to N polarity [9,10]. As a consequence, the growth conditions of yielding electrically active p-type GaN is in very narrow window. In addition, low-hole concentrations are often difficult to reproduce due to the sensitivity of Mg to growth conditions. These complexities lead to variable resistance and mobility of p-GaN. Numerous p-type doping techniques have been developed over the past few decades, including Mg delta doping [11], co-doping [12], superlattice doping [13] and doping with Be [14]. However, none of the mentioned techniques provide a reliable method that can effectively overcome the problem of low-hole concentration in current III-nitride materials. As a result, the state of the art in hole concentration in GaN with Mg doping is generally limited to 1–2 × 10^18^ cm^−3^.

Metal-modulated epitaxy (MME) technology has been reported to greatly increase hole concentration in GaN. MME is a technique in which metal beams (Al, Ga, In, and Mg) are switched on and off in short periods in an RF MBE system, while a continuous nitrogen plasma beam is kept on [15]. We first have to consider the incorporation behavior of Mg and the formation of donor-type compensatory defects at the atomic level, the widely accepted molecular dynamics mechanism for Mg incorporation into the lattice involves “surface aggregation and surface segregation”, which forces Mg to remain at a high concentration on the growth surface and creates Mg-related defects on the growth surface [16,17]. The degree of the surface aggregation and segregation is dependent upon the Mg flux, substrate temperature and III/V ratios [18]. Under N-rich conditions, in which excess N covers the GaN surface, Mg dopants will be preferentially incorporated into Ga substitutional sites on the growth surface, and the N vacancy concentration is reduced due to higher formation energies in N-rich conditions than in Ga-rich conditions. N vacancies act as compensating donors, and the lower the N-vacancy concentration, the higher the hole concentration. The theory of Zywietz et al. [19] shows that Ga on the GaN(0 0 0 1) surface has lower Ga adatom mobility under N-rich conditions, and the Ga diffusion length is significantly shorter. Therefore, the surface extension defects will increase under N-rich condition, and the surface will be rougher. Under Ga-rich conditions, in which the Ga coverage exceeds the N coverage, the incorporation of Mg in the epitaxial layer is reduced due to the reduced number of Ga substitutional sites [20]. Attempts to address this issue with higher Mg fluxes resulted in a local polarity reversal from Ga polarity to N polarity and reduced Mg incorporation. Under Ga-rich conditions, the formation energy of nitrogen vacancies is reduced, so more nitrogen vacancies are available to compensate for free holes [21]. Based on the above analysis, growing p-GaN only under Ga-rich or N-rich conditions is not the best. The best growth method is to cycle between Ga-rich and N-rich conditions. Ga-rich is to prevent the formation of extended defects [22], and N-rich is to promote Mg to Ga substitutional sites, meanwhile reducing the generation of compensatory N vacancies [23,24]. This “oscillation” of growth conditions can be achieved by MME. When the metal source is turned on, metal accumulation occurs on the growth surface, and when the metal source is turned off, the accumulated Ga and Mg on the surface are depleted.

In metal modulation epitaxy, there are three parameters that can be changed to affect the crystal quality of the sample, namely, the fluxes of the metal source, the opening time of the metal source shutter and the closing time of the metal source shutter. According to my literature survey, the previous studies changed one or two of these parameters. In this article, I have studied the influence of three parameters on GaN:Mg films. The effect of periodic duty cyclings on GaN:Mg films (fixed Ga, Mg, N flux) is systematically investigated in this work, and GaN:Mg films with smooth morphology, high crystalline quality and high hole concentration are obtained.

## 2. Experimental

All GaN:Mg films were grown by MME in a GEN 20A MBE system on n-type MOCVD-grown GaN templates, with a chamber pressure of approximately 1 × 10^−10^ Torr. The templates were cleaned using a dilute hydrochloric acid solution in a ratio of 1:3, and then using ultrasonically cleaned with acetone, isopropanol, deionized water in sequence for 10 min each time, and dried with nitrogen before being loaded into a preconditioning chamber. Templates were first degassed in the preconditioning chamber at 200 °C for 60 min, followed by degassing in the growth chamber at 650 °C for 30 min. For all films, nitrogen was supplied by a RF plasma source with the power of 330 W and N_2_ flow of 1.5 sccm. Ga was supplied by a standard effusion cell, and Mg was supplied by the Veeco corrosive valve cracker. During growth, the BEP of Ga was 6.8 × 10^−8^ Torr. An 80 nm thick GaN buffer layer was first grown on templates by regular growth. Then GaN:Mg films were grown at 760 ℃ with the same Ga and Mg flux, but the periodic duty cyclings (open/close) was varied. The total opening time of metal source shutter was 7200 s.

A Veeco Dimension 3100 Atomic Force Microscope (AFM) was used to observe the surface morphology of GaN: Mg samples in tapping mode. The electrical characteristics of the films at room temperature, such as hole concentration and mobility, were determined by a Hall effect measurement device. The samples were cut into small pieces of 1 cm×1 cm, using indium dots as contact electrodes, and the current-voltage curve of each sample was linear to ensure that the contact was ohmic. Mg concentration was detected by secondary ion mass spectrometry (SIMS) measurements. With a high-resolution XRD instrument, the crystalline quality of the films was evaluated by the full width at half maximum (FWHM) of the symmetric (002) and asymmetric (102) diffraction patterns of the rocking curves.

## 3. Results and Discussion

### 3.1. Surface Topography Analysis

**Figure 1:**
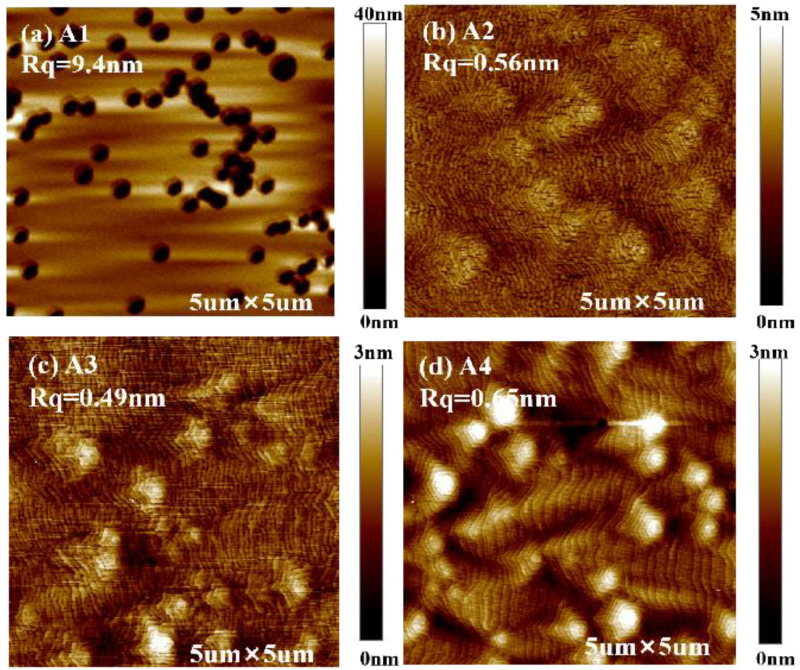
(**a**–**d**) are, respectively, 5 × 5 μm AFM images for sample A1, A2, A3 and A4. The corresponding metal source shutter opening time of samples A1, A2, A3, and A4 are 5 s, 10 s, 15 s, and 20 s, respectively. The metal source shutter closing time is kept at a constant of 5 s.

The metal source shutter closing time was kept constant of 5 s, and the metal source shutter opening time was 5 s, 10 s, 15 s and 20 s, respectively, which are recorded as A1, A2, A3 and A4. Figure 1 shows AFM images for sample A1, A2, A3 and A4 at scan area of 5 × 5 μm, respectively. There are many pits on the surface of sample A1, which appear to be similar to V-shaped defects observed in GaN growth. The surface morphology of A2 and A3 samples are characterized by atomically flat terraces. The RMS roughness is about 0.5 nm. The sample A4 surface is smooth with characteristic spiral growth hillocks. Under this growth condition, the mobility of Ga adatoms is high, and a rich Ga metal layer is formed on the surface, thereby forming a step flow growth mode, and the surface is very smooth. The spiral growth hillocks are considered to comprise double-height steps and terraces due to step flow growth around mixed edge/screw dislocations [25,26]. Since two steps are fixed at each mixed dislocation, the spiral hillock consists of two interlocking spiral ramps [27]. On sample A4, the typical size of the Ga droplets is 3–5 mm at a density of 10^5^ cm^−2^, which is determined by optical microscopy. Longer metal source shutter opening times means higher III/V ratio on average over one cycle, resulting in smoother surfaces, which is consistent with the theoretical considerations reported by Zywietz et al. [19]. The increase of Ga adsorption layer coverage increases the surface adatom diffusion and decreases the surface roughness.

**Figure 2:**
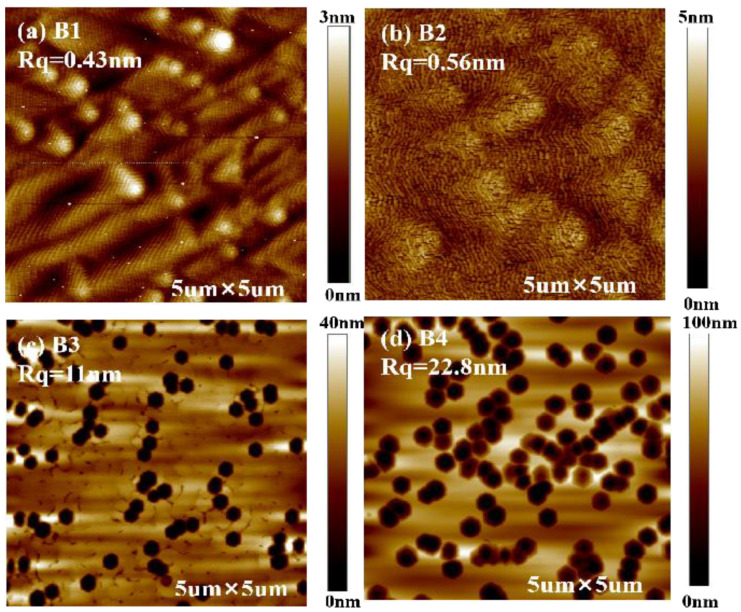
(**a**–**d**) are, respectively, 5 × 5 μm AFM images for sample B1, B2, B3 and B4. The corresponding metal source shutter closing time of samples B1, B2, B3, and B4 are 2.5 s, 5 s, 10 s, and 20 s, respectively. The metal source shutter opening time is kept constant of 10 s.

The metal source shutter opening time was kept constant of 10 s, the metal source shutter closing time was 2.5 s, 5 s, 10 s and 20 s, and the samples are marked as B1, B2, B3 and B4, respectively. As can be seen in Figure 2, the surface morphology of sample B1 is smooth with some spiral hillock features, and even Ga droplets are observed on sample B1 by optical microscopy. The surface morphology of sample B2 is atomically flat terraces, and the RMS roughness is about 0.5 nm. As for sample B3 and B4, the surface is very rough and has a large density of pits, which is due to the too N-rich conditions. As the metal source shutter closing time is increased to 10 s and 20 s, the III/V ratio is small throughout the cycle. The creation of surface pits (depressions) associated with extended defects [28]. The pits in the N-rich region correspond to the sinking and termination of mixed and pure edge threading dislocations (TD). The larger, deeper pits are associated with mixed dislocations, and the shallower pits are associated with edge dislocations.

**Figure 3:**
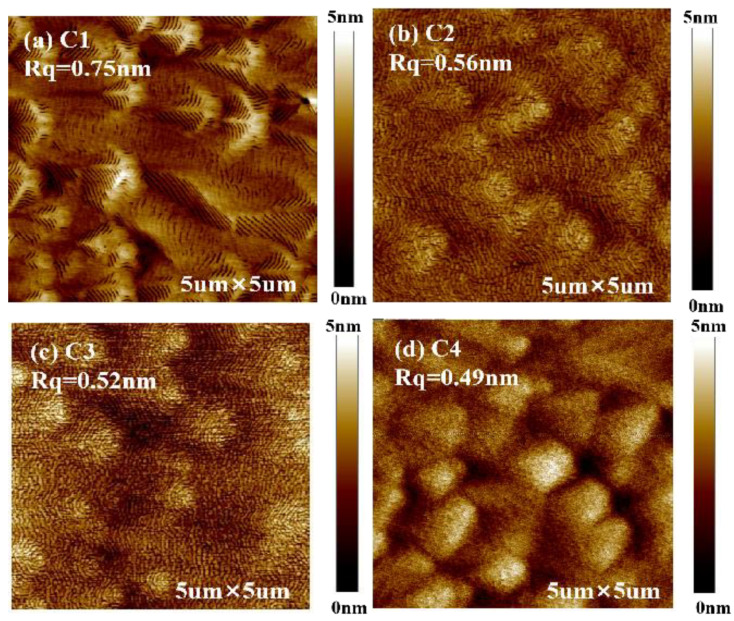
(**a**–**d**) are, respectively, 5 × 5 μm AFM images for sample C1, C2, C3 and C4. The corresponding periodic duty cyclings (open/close) of samples C1, C2, C3, and C4 are 6 s/3 s, 10 s/5 s, 20 s/10 s, and 40 s/20 s, respectively.

We chose four different shutter duty cycles, with duty cycling (open/close) of 6s/3 s, 10 s/5 s, 20 s/10 s, 40 s/20 s, but the total metal source shutter opening time was kept at 7200 s. The samples were denoted as C1, C2, C3, and C4 respectively. As shown in Figure 3, smooth surface with characteristic spiral growth hillocks can be seen in the four samples. The roughness is similar except that sample C1 is a little rougher. We believe that this is because their average V/III ratios are the same.

### 3.2. SIMS and Hall Analysis

**Figure 4:**
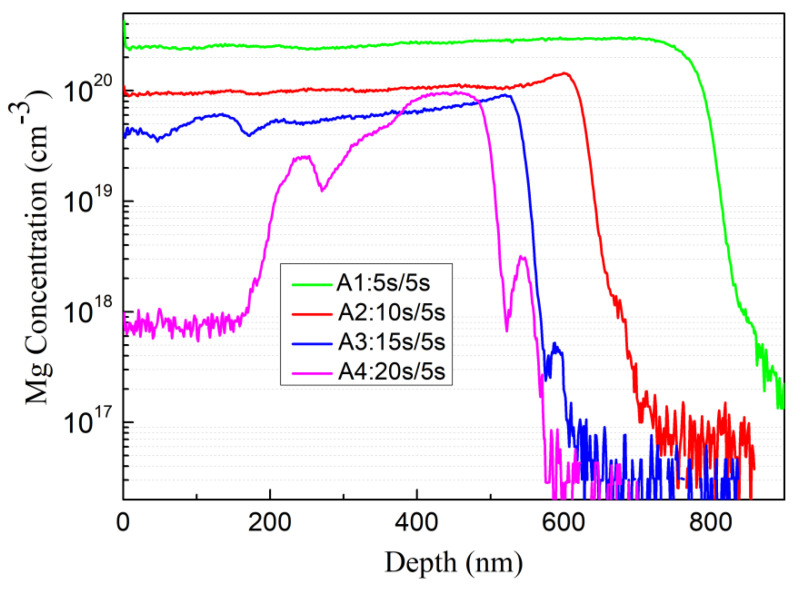
SIMS profile, Mg doping concentration of sample A1, A2, A3 and A4.

According to the SIMS measurement, the Mg doping concentrations of the four samples A1, A2, A3, and A4 are 2.5 × 10^20^ cm^−3^, 1 × 10^20^ cm^−3^, 6 × 10^19^ cm^−3^, and 1 × 10^18^ cm^−3^, respectively, which can be seen from Figure 4. There is a sharp drop in the Mg concentration in the A4 sample, which we speculate is because that the growth surface is enriched with excess metal droplets, hindering the incorporation of Mg. However, only sample A2 and A3 appear as p-type, and the hole concentration is 2 × 10^18^ cm^−3^ and 1.4 × 10^18^ cm^−3^_,_ respectively. As there are too many compensation defects in A1, although the Mg doping concentration of sample A1 is the highest, it appears as n-type probably due to the compensatory defects. Due to the excessive metal droplets on the A4 surface, the incorporation of Mg into the lattice is inhibited under too Ga-rich conditions. The doping concentration of Mg on the surface is only 1 × 10^18^ cm^−3^, and the generated holes are too few to offset the influence of intrinsic defects, so it appears n-type in the Hall test.

**Figure 5:**
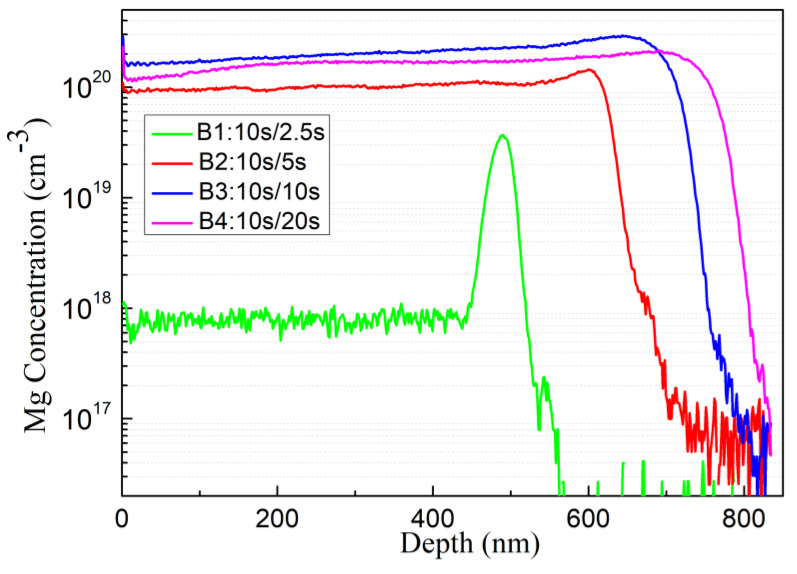
SIMS profile, Mg doping concentrations of sample B1, B2, B3 and B4.

According to the SIMS measurement, as shown in Figure 5, the Mg doping concentrations of the four samples B1, B2, B3, and B4 are 8 × 10^17^ cm^−3^, 1 × 10^20^ cm^−3^, 2 × 10^20^ cm^−3^, and 1.8 × 10^20^ cm^−3^, respectively. However, only sample B2 appears as p-type, and the hole concentration is 2 × 10^18^ cm^−3^. The other three samples appear as n-type GaN. As the metal source shutter closing time becomes longer, the overall average III/V ratio in the period decreases, and the Mg doping concentration generally increases. The excessively long shutter closing time for sample B4 results into slightly reduced Mg concentration compared to sample B3 probably due to aggravated Mg desorption. Thus, B1 appears as n-type mainly because Mg concentration is too low, and B3 and B4 are attributed to high density compensatory defects.

**Figure 6:**
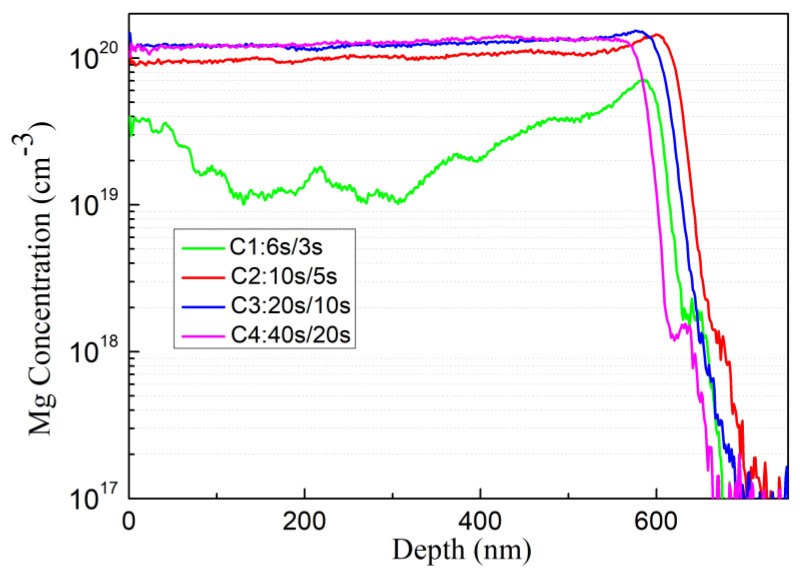
SIMS profile, Mg doping concentration of sample C1, C2, C3 and C4.

The results of SIMS measurements shown in Figure 6 indicates that the Mg doping concentrations of the four samples C1, C2, C3, and C4 are 3 × 10^19^ cm^−3^, 1 × 10^20^ cm^−3^, 1.2 × 10^20^ cm^−3^, and 1.2 × 10^20^ cm^−3^, respectively. Except for C1, the Mg doping concentration is about the same. This is because the III/V ratios of the latter three samples remain the same. C1 may be due to the fact that the metal source shutter closing time is too short. Therefore, Ga and Mg atoms do not have enough time to diffuse and migrate on the growth surface, and find a suitable lattice position for incorporation, so the Mg doping concentrations is reduced.

**Figure 7:**
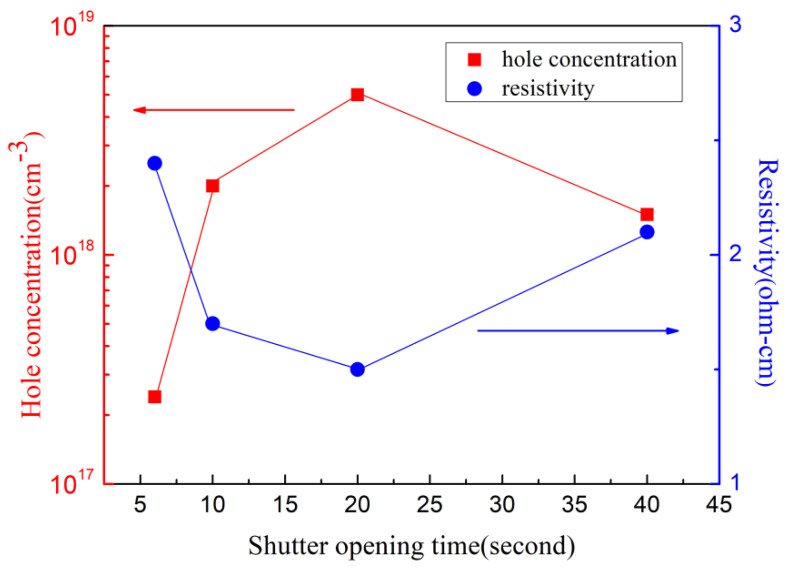
Comparison of hole concentration and resistivity vs metal source shutter opening time.

Figure 7 shows the variations of the hole concentration and the resistance with the metal source shutter opening time, according to the Hall test results of the four samples. It can be found that the hole concentration increases at first and then decreases, and the resistance shows the opposite tendency with the increasing opening time. A high hole concentration of 5 × 10^18^ cm^−3^ is obtained; for comparison, the highest hole concentration of typical p-GaN in the world is 1–2 × 10^18^ cm^−3^, which is a great progress. However, hole mobility is relatively low, about 2 cm^2^/V·S. This is probably due to the high doping concentration of Mg, which forms a lot of point defects and extended defects, causing serious ionized impurity scattering. A low resistivity of 1.5 Ω·cm is obtained by optimizing the metal source shutter opening time. Since Mg doping concentrations are almost the same for the other three samples, the increase in hole concentration is mainly due to the incorporation of surface accumulated Mg dopants into suitable Ga substitutional sites, while the formation of compensatory defects (N vacancies and extended defects) is minimal.

### 3.3. X-ray Diffraction Analysis

**Figure 8:**
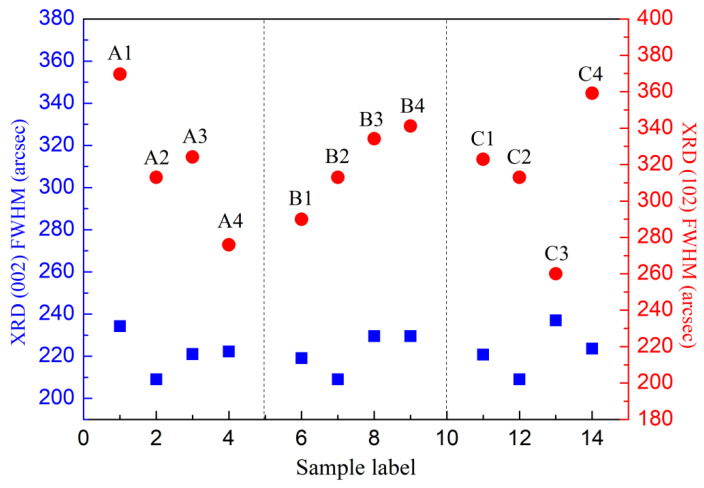
The full width at half maximum of (002) plane and (102) plane of three groups of samples.

We measured the X-ray diffraction rocking curve of these three groups of samples and obtained full width at half maximum (FWHM) of (002) plane and (102) plane, as shown in Figure 8. Screw dislocation density and edge dislocation density in the GaN material can be estimated by the FWHMs of GaN (002) and (102) plane rocking curve [29]. The (002) plane FWHMs of these three groups of samples are basically between 210-230 arcsec, with little difference. The (102) plane FWHMs of sample C3 is 260arcsec, apparently lower than those of other samples. The screw dislocation density and the edge dislocation density of C3 were calculated as 1.42 × 10^8^ cm^−2^ and 3.86 × 10^8^ cm^−2^, respectively. The sample C3 shows the lowest edge defect density and the least defect compensation, which is consistent with the highest hole concentration obtained by the Hall test.

## 4. Conclusions

In summary, we systematically studied the effect of the periodic duty cycling on the surface morphology, crystal quality, Mg doping concentration, and electrical properties of Mg-doped GaN films grown by MME technique. It was found that when the BEP of Ga was 6.8 × 10^−8^ Torr, nitrogen was supplied by a RF plasma source with the power of 330 W and N2 flow of 1.5 sccm, the substrate temperature is 760 ℃, the metal shutter duty cycling is 20 s open/10 s close, and the sample has a high Mg doping concentration of over than 1 × 10^20^ cm^−3^ with a smooth surface. The RMS roughness is about 0.5 nm. The FWHM of (002) XRD rocking curve is 230 arcsec and the FWHM of (102) XRD rocking curve is 260 arcsec. As a result, a hole concentration of 5 × 10^18^ cm^−3^, corresponding Mg activation rate of 4.2%, and a resistivity of 1.5 Ω·cm have been obtained, which is important to develop optoelectronic device such as laser. The hole concentration increases due to the incorporation of surface accumulated Mg dopants into suitable Ga substitutional sites with minimal formation of compensatory defects.

## Data Availability

Not applicable.
